# Absorption of Fats

**Published:** 1891-06-20

**Authors:** 


### THE ABSORPTION OF FATS.

The value of fat as a food renders a clear appreciation of the physiological processes by which it is absorbed of the greatest importance. Like the proteids and the carbohydrates, fat acts as a valuable source of potential energy, and its percentage of potential energy is higher than that of either of these two classes of food stuff, inasmuch as it contains a larger proportion of oxidisable material. It was till recently held that the mechanical phenomena of osmosis explained the method of its absorption, but it has been sufficiently demonstrated that inexplicable vital factors are necessary to the process. The fat particles are probably seized by pseudopodia projected from the living epithelium of the cœlli of the intestines, especially of the duodenum and of the upper portions of the jejunum. But for this selective process on the part of the intestinal epithelium to take place the fat must be presented in a suitable form as an emulsion. Bernard discovered that by a special fermentative process the pancreatic juice fats in the intestines were partially hydrated. Fats are chiefly trivalent alcohols united with glycerine and fatty acids. By hydration the glycerine and fatty acids are liberated. If only a small portion of the fat undergoes hydration, the remainder is at once emulsified and the alkaline salts of the bile saponifies this emulsion, the intestinal juices probably also taking part in the saponification of fat. The functions of the pancreas in relation to fat absorption have been investigated by Abelmann and by Minkowski. They found that in dogs totally deprived of the pancreas, when meat and bread with milk were given in such quantities as to supply 7 to 24 grammes of nitrogen daily, 44 per cent. underwent absorption. In dogs in which a small portion of the gland remained, on an average 54 per cent. was absorbed. Moreover, the addition of fresh pancreas to the diet caused an immediate rise in the amount of nitrogen absorbed to about 75 per cent. of that ingested. In those cases in which the whole of the pancreas had been removed, and in which nearly the whole of the fats were discharged unabsorbed, they appeared to the extent of about four-fifths in the state of fatty acids and partly in the form of soaps.

In addition to the action of the bile in aiding absorption by the saponification of fats, it has been shown by Thanhoffer that the activity of the protoplasmic processes of the intestinal epithelium become more marked when wetted with bile. Dr. Caldwell, of Chicago, has considered this question of fat absorption in a recent lecture, and points out that the millions of villi in the duodenum and jejunum constitute the *mechanism*. Each villus consists of a conical projection covered by a single layer of columnar epithelium on a basement membrane, beneath which and occupying the villus is a spongy network of admoid tissue, whose inter-communicating channels extend from the lymph spaces beneath the epithelium to a central lacteal. This lacteal has valves opening centreward, and around it, extending from apex to base, are arranged longitudinally unstriped muscular fibres. A small artery is placed excentrically in each villus. The saponified fatty molecule is seized, carried through the columnar epithelium into the lymph spaces, and there it enters the white corpuscles, and passes along the lymph channels to the central lacteal, whence it is carried down to the chyle vessels by the contractions of the muscle fibres referred to, and on to the thoracic duct. Although a portion of the fat is hydrated and split into fatty acids and glycerine and absorbed in this form, the greater portion remains unchanged. The other and smaller portion absorbed as fatty acids and glycerine, are, after absorption, reunited again to form neutral fat, this process taking place in the intestinal tissues. Ewald found that fat was formed from soap and glycerine in the presence of intestinal mucous membrane. Munk has shown that in dogs fed on fatty acids a large proportion of neutral fat appears in the chyle, and in his experiments only a small proportion of the fatty acids absorbed appeared in the chyle as unaltered fatty acids. The action of the pancreas appears to greatly aid in the absorption of fat, but more important still is the action of the bile. Experimental research has confirmed clinical experience that it is useless to administer fat as food if the liver is not properly performing its function, and the extreme importance of the attention to the state of the liver in wasting diseases becomes obvious.

